# “Today I Can Look in the Mirror and Like Myself”: Effects of a Trauma-Informed Mindful Recovery Program on Self-Compassion

**DOI:** 10.3389/fpsyg.2022.780383

**Published:** 2022-06-02

**Authors:** Sarah K. Moore, Kayley Okst, Lydia Smith, Thomas Fatkin, Timothy Creedon, A. Kiera Fredericksen, Richa Gawande, Zev Schuman-Olivier

**Affiliations:** ^1^Center for Technology and Behavioral Health, Geisel School of Medicine at Dartmouth College, Hanover, NH, United States; ^2^Center for Mindfulness and Compassion, Cambridge Health Alliance, Cambridge, MA, United States; ^3^Department of Psychiatry, Harvard Medical School, Boston, MA, United States

**Keywords:** mindfulness, opioid use disorder, buprenorphine, self-compassion, trauma-informed, mixed method

## Abstract

**Background:**

Opioid-related deaths continue to rise. Psychological trauma is commonly comorbid with Opioid Use Disorder (OUD). Adverse childhood experiences can disrupt the development of emotion regulation, increasing risk of substance use. Self-compassion may reduce OUD risk and outcomes by facilitating emotion regulation, decreasing the toxicity of shame, and reducing internalized stigma that can hinder recovery. Mindfulness practice enhances self-compassion.

**Methods:**

This study is part of a pilot (*N* = 18) of the Mindful Recovery OUD Care Continuum (M-ROCC) during buprenorphine office-based opioid treatment (OBOT). The present study was conducted to gain a deeper understanding of the intervention’s effects on self-compassion development, and to explore differential changes in self-compassion during the intervention among participants with varying intensity of trauma exposure measured by high levels of childhood adversity (defined by 4+ adverse childhood experiences (ACEs) at baseline). We conducted secondary analyses of a subset of qualitative interview data (*N* = 11 unique participants) collected for the pilot study (weeks 4 and 24, 14 total interviews) to elaborate upon changes in Self-Compassion Scale (SCS-SF) scores.

**Results:**

In the primary pilot study, participants’ mean SCS-SF scores shifted significantly from baseline to week 24, *β* = 0.22, *p* = 0.028. This change is elaborated upon through interviews. Despite pervasive challenges to becoming more self-compassionate (e.g., trauma histories and substance use), participants reported increased compassionate self-responding and decreased uncompassionate self-responding. Mindfulness training was identified as the primary mechanism underlying the shift. Kindness to self and others and—to a lesser extent an increased sense of common humanity—were also identified as key to overall self-compassion. Compared to those in the lower ACEs group, participants in the higher ACEs group tended to have lower baseline self-compassion scores (*d* = 1.09, *p* = 0.055).

**Conclusion:**

M-ROCC may increase self-compassion among patients with OUD during OBOT by increasing compassionate, and decreasing uncompassionate, self-responding. Patients with OUD with greater childhood adversity tended to have lower levels of self-compassion, which improved with M-ROCC. Future trials with larger samples are needed to confirm these potential outcomes, mechanisms, and differential impacts between ACEs subgroups.

## Introduction

Drug overdose deaths in the United States increased by 30.9% between January 2020 and January 2021 (Ahmad et al., 2021). Demonstrating the pervasiveness of this crisis, over 35 states recorded spikes in opioid-related mortality since the beginning of the global COVID-19 pandemic (AMA, 2020). Suicide rates have also increased significantly over the past two decades (Rizk et al., 2021), and while all substances elevate the risk for suicidal behavior, alcohol and opioids are the most common substances identified in suicide decedents (Esang and Ahmed, 2018).

The landmark Adverse Childhood Experiences (ACEs) study demonstrated the link between exposure to childhood trauma and adult risk behaviors, including illicit drug use (Felitti et al., 1998). An ACE score (range: 1–10; e.g., parental divorce) has been used as a tool for describing the population impact of the cumulative effect of childhood stress, providing a framework for understanding how prevention of ACEs can reduce the burden of many public health problems and concerns. Childhood trauma and ACEs have been reported to be very common in the histories of those with substance use disorders and opioid use disorder (OUD), specifically. Rates of childhood psychological trauma were disproportionately high among those living with substance use disorders (SUDs; Heffernan et al., 2000; Naqavi et al., 2011), including individuals living with OUD (Moran et al., 2018; Gannon et al., 2021), and may potentiate the development of SUDs and decrease quality of physical and mental health for those living with OUD. For example, opioid-dependent individuals with histories of sexual abuse had poorer mental and physical health and have been found to be heavier users of opioids as compared to opioid-dependent individuals with no history of sexual abuse (Heffernan et al., 2000; Bartholomew et al., 2005; Charney et al., 2007).

Opioid use has been theorized to “numb” unwanted emotional states, especially in cases where self-regulation during childhood was impaired in some way (Hien et al., 2005). Self-medication occurs in a context of suffering from self-regulation deficits (Khantzian, 1997; Khantzian and Albanese, 2008), such as difficulties in regulating emotions, sustaining interpersonal relationships, and maintaining self-care. In particular, adverse experiences during childhood create vulnerabilities that have the potential to interfere with the normal development of emotion regulation (Kalia and Knauft, 2020). A recent study of individuals with OUD who were in methadone maintenance treatment extended the evidence supporting an association between impaired emotion regulation and coping motives—previously found in alcohol and marijuana users—to OUD (Gold et al., 2020).

In addition to the increase in research and development of mindfulness-based interventions, many believe that mindfulness and compassion are linked, and that compassion may be one implicit ingredient of mindfulness (Hölzel et al., 2011). Over the last two decades, there has also been a marked increase in compassion-based research and interventions that support cultivation of compassion for others and self (Kirby et al., 2017). Self-compassion—compassion turned inward or a kind way of relating to ourselves in instances of perceived failure, inadequacy, or personal suffering (Neff, 2016)—and its benefits have been shown to be protective for mental health (e.g., reduce anxiety and depression (Van Dam et al., 2011), substance use (Li et al., 2017; Goldberg et al., 2018), and general wellbeing (Baer et al., 2012; Phelps et al., 2018). They have also been shown to improve coping and increase resilience (Brion et al., 2014; Zhang and Chen, 2016, 2017), reduce the stress response (Arch et al., 2014; Breines et al., 2014, 2015), and be positively associated with improved emotion regulation abilities (MacBeth and Gumley, 2012). A growing body of literature also demonstrates the protective capacity of self-compassion following traumatic exposure (Thompson and Waltz, 2008; Zeller et al., 2015). Conceptually, self-compassion may function to offset the formation of self-critical or shame-based assumptions (e.g., “I’ll never get better” and “I beat myself up for my past”), according to Germer and Neff (2015). Notably, compassion-focused therapies grew out of increasing recognition that the relationships we have with ourselves, particularly in the forms of shame (Kim et al., 2011) and self-criticism (Kannan and Levitt, 2013), underpin a wide range of mental health issues (Gilbert and Irons, 2005)—suggesting implications for targeting substance use and related shame (Phelps et al., 2018).

Self compassion “involves being open to and moved by one’s own suffering, experiencing feelings of caring and kindness toward oneself, taking an understanding, non-judgmental attitude toward one’s inadequacies and failures, and recognizing that one’s own experience is part of the common human experience” (Neff, 2003a, p. 224). Neff (2003a,b) developed the widely used Self-Compassion Scale (SCS), which has been validated across many different groups, languages, and cultures (e.g., Arimitsu, 2014; Coroiu et al., 2018; Halamová et al., 2018; Neff et al., 2019, 2021). The SCS is multidimensional and includes three subscales that represent compassionate self-responding (CSR)—(1) self-kindness (the ability to treat oneself with care rather than harsh self-judgment), (2) common humanity (the recognition that imperfection is a shared aspect of the human experience, rather than feeling isolated by one’s failures), and (3) mindfulness (the holding of one’s experience in balanced perspective rather than overidentification with suffering). There are an additional three subscales that represent the antitheses of CSR: uncompassionate self-responding (USR)—(1) self-judgment (harshly criticizing oneself for one’s failings), (2) isolation (feeling abnormal and alone in the experience of suffering), and (3) overidentification/self-absorption (fused with suffering such that perspective is lost; Neff, 2020). The three subscales represent three distinct dimensions to self-compassion: self-kindness and self-judgment refer to *emotional* ways that people respond to suffering; common humanity and isolation refer to ways people *cognitively understand* their suffering; and mindfulness and overidentification refer to how people *pay attention* to their suffering. In her work, Neff conceptualizes self-compassion as a dynamic system comprised of these six distinct but interrelated elements:

*“Self-compassion involves simultaneously engaging in compassionate self-responding and disengaging in uncompassionate self-responding…learning to be more self-compassionate reduces the tendency to judge myself, to feel isolated from others, or to become absorbed in negative emotions such as shame”* (2020).

Recently, an efficient alternative to the SCS has been validated with the same factor structure, good internal consistency, and a near-perfect correlation with the SCS, the Self-Compassion Scale-Short Form (SCS-SF; Raes et al., 2011).

Interventions that enhance mindfulness and self-compassion may be helpful for treating people with OUD. Compassion-focused therapies assume the human brain is susceptible to self-absorption, negativity bias, and self-critical self-monitoring (Baumeister et al., 2001; Gilbert, 2009). These therapies developed initially to help people create warmer feelings toward themselves and to help them cultivate a more compassionate way of talking to themselves. For therapists, using a compassion-based therapy involves enabling individuals to develop self-compassion, compassion toward others, and openness to compassion from others, in particular in response to adversity or threatening situations Leaviss and Uttley, 2015). People can learn to respond to suffering (e.g., self-shaming) in a soothing, healing way through self-compassion (Germer and Neff, 2015). This is particularly relevant in a population with OUD where people who use opioids often feel large amounts of shame and internalized stigma (Yang et al., 2019)—both currently around their drug use and historically due to trauma (Carlyle et al., 2019). Unfortunately, previous research investigating the effects of adding behavioral interventions to buprenorphine treatment *have not* consistently reported beneficial impacts (Carroll and Weiss, 2017). However, pilot studies that have added specific mindfulness-based components to medication treatment for OUD (MOUD) have been shown to be feasible and acceptable (Price et al., 2020; Fatkin et al., 2021) and have reported significant potential benefits, such as decreased substance use and chronic pain (Garland et al., 2022) and increased interoceptive awareness (Price et al., 2020), warranting further investigation.

Learning to be more self-compassionate may reduce the tendency to judge oneself, feel isolated and disconnected from others, or become absorbed in negative emotions, such as shame (Neff, 2020). Self-compassion is notably both a challenge and an opportunity for trauma survivors (Germer and Neff, 2015) and may be a key mechanism of change underlying the power of mindfulness [“the awareness that emerges through paying attention on purpose… nonjudgmentally, to the unfolding of experience moment to moment” (Kabat-Zinn, 2003)] practice to decouple negative thinking from positive outcomes (Kuyken et al., 2010). Even 2 weeks of self-compassion training can lead to reduction in self-criticism and demonstrable changes in neural activity during periods of self-criticism (Lutz et al., 2020). Given the high rates of self-criticism (Carlyle et al., 2019) and a proposed deficit of self-compassion in patients with OUD (Phelps et al., 2018), mindfulness and self-compassion-based interventions may be particularly advantageous. Overall, self-compassion is a demonstrably learnable skill with many potential applications in psychology and related fields (Germer and Neff, 2013) and is worth exploring in an OUD population.

This pilot study is part of a larger investigation of the feasibility and acceptability and the effects of the Mindful Recovery Opioid Use Disorder Care Continuum (M-ROCC) intervention on comorbid anxiety, pain, and substance use during buprenorphine treatment in primary care. M-ROCC is adapted from Mindfulness Training for Primary Care, which is a trauma-informed, “warm” mindfulness-based program, which explicitly weaves in threads of warmth and self-compassion in each session. M-ROCC is a recovery program based on MTPC that is designed to be flexibly implemented in primary care settings (Fatkin et al., 2021; see methods for a more in-depth description of M-ROCC). A primary target of this research program was identifying the influences of mindfulness meditation on self-regulation mechanisms involved in behavior change and measuring their effect size for powering a randomized controlled trial. One key mechanistic domain of self-regulation was self-related processes like self-compassion (Schuman-Olivier et al., 2020). The single-arm feasibility and acceptability study of M-ROCC (*N* = 18) showed that 89% of participants (*n* = 16) were retained at week 4, 72% (*n* = 13) were retained at week 24, and 100% would refer a friend (*n* = 18; Fatkin et al., 2021). The present mixed methods, secondary analysis was conducted to gain a deeper understanding of the effects of M-ROCC on the development of self-compassion, and to explore how changes in self-compassion during M-ROCC among participants with “higher” (>/= 4) and “lower” (<4) adverse childhood experiences may be differentially impacted by the mindfulness intervention.

## Materials and Methods

### Study Design

We implemented the M-ROCC intervention in a feasibility and acceptability pilot study at two Cambridge Health Alliance (CHA) primary care sites (Fatkin et al., 2021) and examined preliminary health outcomes and self-regulation mechanisms (manuscript under review). This mixed methods, secondary analysis study examines a salient outcome of the primary feasibility and acceptability study: statistically and clinically significant changes from baseline to week 24 were observed for the total self-compassion score on the SCS-SF. We used a convergent parallel mixed method design for the purpose of complementarity—“seek[ing] elaboration, enhancement, illustration, clarification of the results from one method with the results of the other method” (Schoonenboom and Johnson, 2017, p.110). Thus, we sought elaboration of these preliminary findings and the development of self-compassion in this context through an exploration of relevant qualitative interview data, rather than the SCS-SF subscales. Operationalizing these outcomes using the SCS-SF subscales is not recommended, as they are considered less reliable than the total score on the short form (Raes et al., 2011). Additionally, as the M-ROCC intervention is trauma-informed, we sought to explore changes in self-compassion during the M-ROCC intervention among participants with “higher” (>/= 4) and “lower” (<4) adverse childhood experiences at baseline.

Self-compassion (SCS-SF) was assessed at baseline, 4, and 24 weeks, and trauma history (ACEs) was assessed at baseline. Qualitative interviews were conducted at 4 and 24 weeks with interested participants. All survey data were recorded through REDCap data capture tools (Harris et al., 2009) either directly by participants, or by members of the research team. We recruited participants from two primary care OBOT sites on an ongoing basis between December 2018 and July 2019 *via* direct referrals from providers and/or a study flyer. Of the 25 recruited, *N* = 18 passed eligibility requirements and completed baseline measures, making them eligible to begin the intervention. This study was approved by the CHA Institutional Review Board, published on ClinicalTrials.gov (NCT 03798431), and had an NCCIH-approved data safety monitoring plan with an independent monitoring committee. For further details regarding study recruitment, consent, screening, baseline assessments, and reimbursement structure, please refer to Fatkin et al., 2021.

### Intervention: Mindful Recovery OUD Care Continuum (M-ROCC)

**Figure 1:**
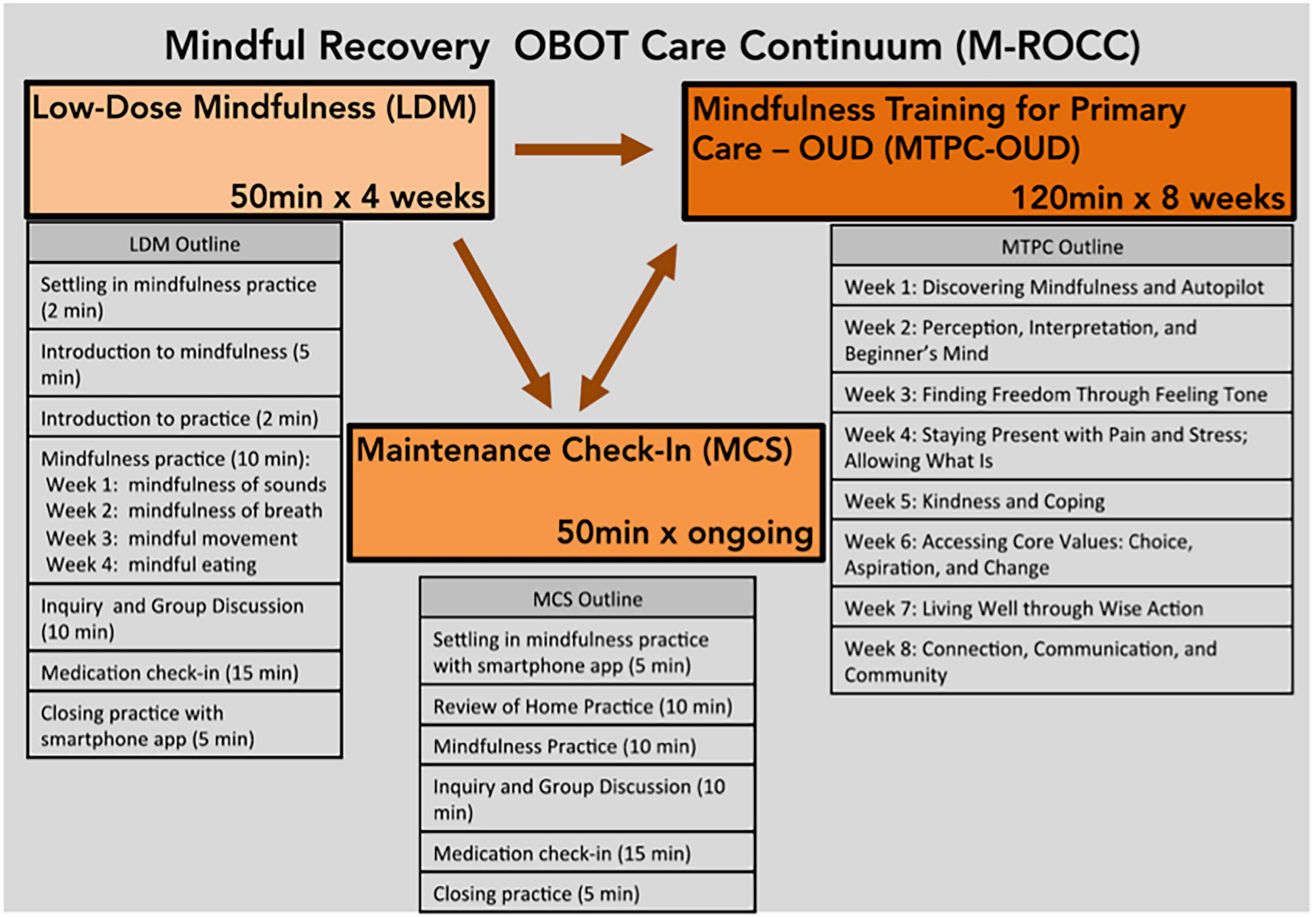
M-ROCC flow diagram.

M-ROCC is a stage-oriented, motivationally sensitive, trauma-informed mindfulness program that aims to support patients in addiction recovery to cope with stress, anxiety, depression, cravings, pain, and other addictive behaviors. The intervention embodies key principles of mindfulness, such as present moment awareness, acceptance of one’s experiences, non-judgment, and self-compassion. M-ROCC is motivationally sensitive, since participants are given a choice about how and when they moved through the various stages of the program: Low-Dose Mindfulness (LDM), Mindfulness Training for Primary Care-OUD (MTPC-OUD), and Mindfulness Maintenance Check-In Support (MCS; See Figure 1 for M-ROCC flow diagram). The program is designed to gradually introduce participants to mindfulness, incorporating experiential inquiry-based learning and practices, and to allow participants to move through these stages as they feel ready to avoid feeling overwhelmed (e.g., a participant can choose to repeat LDM, move directly into MCS and skip MTPC-OUD altogether, and move from LDM to MTPC-OUD to MCS sequentially). During the 4-week LDM sessions, participants are slowly introduced to key mindfulness principles and practices, including mindfulness of sounds, breath, movement, and eating. Participants are encouraged to practice the skills they learn in the group at home, building confidence and endurance through an ascending practice dose ladder (e.g., starting with 5 min of practice per day and building to 20 min per day over the 4-week period). The 8- or 16-week MTPC-OUD curriculum expands upon the foundational skills learned in LDM (e.g., recognizing when participants are on “autopilot” in their everyday lives, cultivating kindness and warmth toward oneself) and also encourages increased home practice. MCS provides a group environment for participants to continue fostering their mindfulness practices in a supportive group environment. The intervention is trauma-informed (CSAT, 2014) as it allows participants a large degree of choice—in the groups they join, how they participate in group practices, and the amount they practice at home. Participants also learn to become aware of their conditioned trauma responses and to minimize avoidance and reactivity to those responses through psychoeducation and inquiry, a technique used in MBPs to allow participants to explore their moment-to-moment experiences as they arise. In “warm” mindfulness training, this can also allow for compassionate connection with the group leaders and other participants. MTPC is a mindfulness-based program (MBP) that follows the traditional MBP structure (Crane et al., 2017), while also cultivating self-compassion, warmth, and kindness as a core thread that is woven into every session. For the specific kindness/compassion practices, the fourth week of LDM included a compassionate body scan and a self-compassion medication taking practice. The fifth week of the 8-week MTPC included a kindness meditation, a self-compassion break, and the giving and receiving compassion practice. The M-ROCC program was also designed to be flexibly implemented in primary care settings, and, as such, the MTPC-OUD portion, which was adapted from the original MTPC curriculum (Gawande et al., 2019) to orient toward addiction recovery, can be delivered in either an 8- or 16-week format. All M-ROCC groups were facilitated by licensed clinicians with experience in office-based opioid treatment and were MTPC-trained group leaders. For additional information regarding intervention details, please refer to Fatkin et al., 2021.

### Quantitative Outcomes and Analysis

The *ACE Questionnaire* includes detailed questions about adverse childhood experiences (e.g., emotional, physical, or sexual abuse) and family and household dysfunction (e.g., intimate partner violence, mental illness in parents, or other household members). The questionnaire consists of 10 yes-or-no items (e.g., “Did a parent or other adult in the household often push, grab, slap, or throw something at you?”). Higher scores indicate more ACEs. The ACE is a reliable (Cronbach’s alpha = 0.88: Murphy et al., 2014) and valid screen for retrospective assessment of adverse childhood experiences (Wingenfeld et al., 2010). The *Self-Compassion Scale Short-Form* includes 12 items across 6 subscales of self-compassion: self-kindness, self-judgment, common humanity, isolation, mindfulness, and overidentification (Raes et al., 2011). The response scale ranges from 1 (almost never) to 5 (almost always). To calculate a total self-compassion score’ mean, the negative subscale items were reversed, with higher scores denoting greater levels of self-compassion (Cronbach’s alpha = 0.86 calculated with current study data).

Descriptive statistics were used to examine demographic characteristics as well as participant attendance, survey completion, and recruitment. To measure change in SCS-SF scores over time, we conducted linear mixed models in a repeated measure analysis. Due to its small sample size, this study was not powered to detect statistically significant differences between baseline and week 24 time periods. Only participants who began the intervention (*n* = 18) were included in analyses [mixed model approach for intent-to-treat analyses to account for missing data (Chakraborty and Gu, 2009)], defined as attending at least one M-ROCC session. ACE scores were dichotomized (0–3: lower trauma/4+: higher trauma) due to the high rate of chronic health problems among individuals with a score of 4 or more (Felitti et al., 1998). Linear mixed models repeated measure analyses were performed to measure change in SCS-SF scores from week 0–24 for the full sample and between ACEs groups. To prevent bias during analysis, statistical consultants (LS & TC) conducted the analysis in STATA 16.1.

### Qualitative Interviews and Analysis

**Figure 2:**
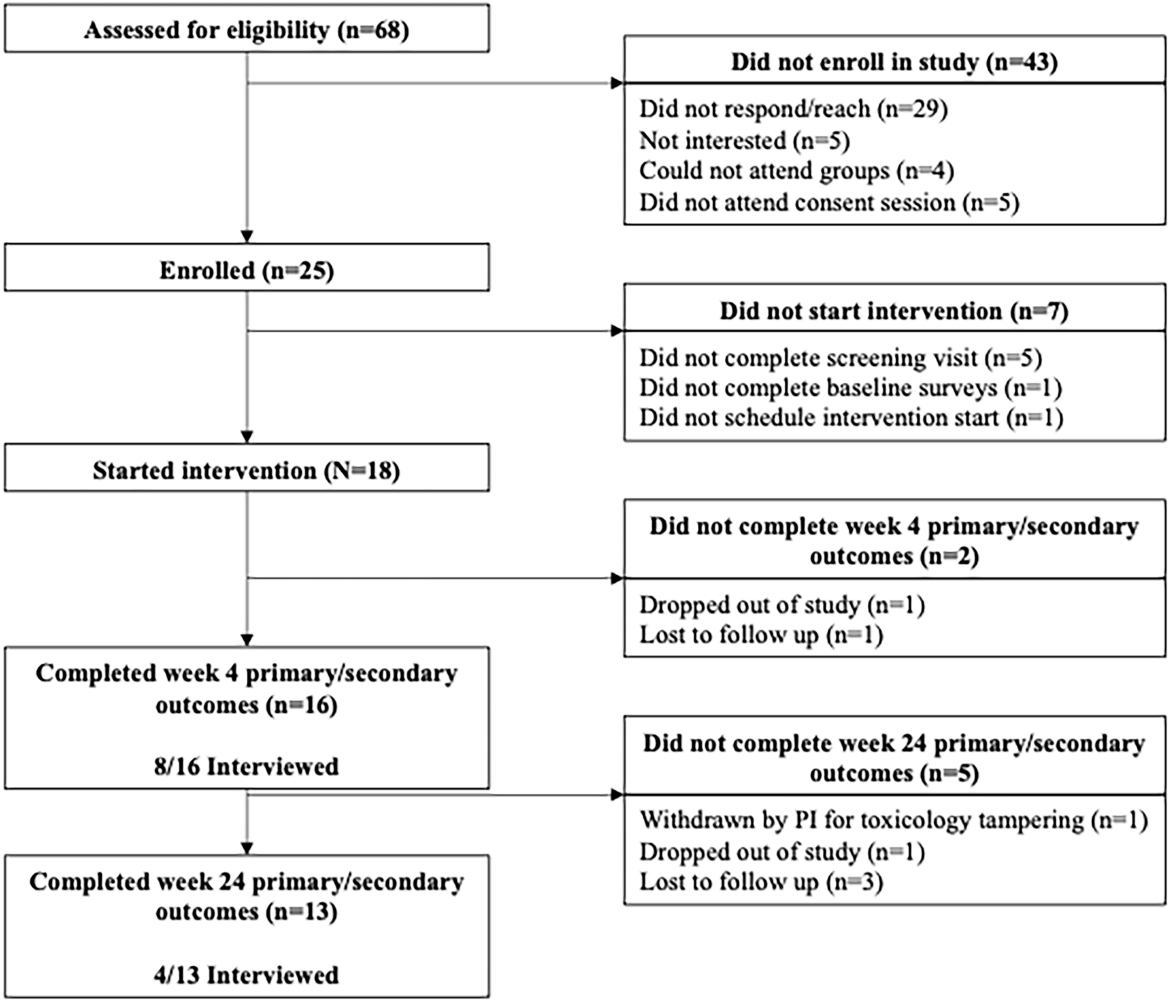
Consort diagram.

During the larger mixed methods investigation of the feasibility and acceptability of the M-ROCC intervention, qualitative interviews with 12 unique (several participants were interviewed twice; however only 11 participant interview transcripts included text coded relevant to study aims; See Table 1 for more detail) participants were conducted at week 4 (*n* = 12) and week 24 (*n* = 4) to explore participants’ experiences with the stage-oriented continuum of care (See Figure 2 for Consort Diagram). The opportunity to be interviewed by an experienced qualitative interviewer and methods expert unrelated to the intervention team (SM; *via* Zoom video conference) was extended to all participants interested in providing feedback on their experiences in the program. Interviews were audio- and video-recorded on Zoom software (Zoom Video Communications Inc, 2016) and transcribed through Rev.com Inc. transcription services (Rev.com, 2021). Transcripts were uploaded to Atlas.ti (8.4; Muhr, 2019) for content analysis (Hsieh and Shannon, 2005). Two themes relevant to self-compassion emerged during the feasibility/acceptability study—“lovingkindness toward self” and “applications of mindfulness in everyday life”—and thus, the relevant coded text were deemed suitable for a more in-depth subtheme analysis and are the focus of this substudy. A qualitative methods trainee (KO) and a qualitative methods expert (SM) independently reviewed all transcripts, collaboratively developed an iteratively revised codebook based on analyses of small batches of data, and finally independently coded the bulk of the data with the revised codebook. Agreement between coders was achieved through discussion. Coding of the data related to the above themes was a hybrid of deductive (i.e., 6 subscale factors of the SCS-SF, e.g., self-kindness and isolation) and inductive (i.e., codes surfaced during analysis related to self-compassion: challenges of self-compassion, long history/past, trauma/substance use related shame, compassion toward others, M-ROCC as causal, and process of becoming compassionate). Methodologists met weekly until all reports were discussed, and subthemes were collaboratively identified (refer to Fatkin et al., 2021 for more detail on the qualitative analyses).

**Table 1:** Participant interview participation and exposure to M-ROCC.

ID Number	Week 4 Interview	Week 24 Interview	Number of Groups Attended/Number of Groups Scheduled to Attend
01	Y	Y	10/10 weeks LDM, 8/8 weeks MTPC, 6/6 weeks repeated MTPC
02	Y	Y*	8/10 weeks LDM, 7/8 weeks MTPC, discontinued intervention
03	Y		8/10 weeks LDM, 6/8 weeks MTPC, 2/2 weeks MCS, discontinued intervention
04	Y		4/6 weeks LDM, 6/8 weeks MTPC, 7/10 weeks MCS
05	Y		13/19 weeks LDM, 4/5 weeks MCS
06			
08	Y		15/16 weeks LDM, 4/4 weeks MTPC, discontinued intervention
09	Y		4/6 weeks LDM, 7/8 weeks MTPC, 9/10 weeks MCS
10	Y	Y	10/11 weeks LDM, 7/8 weeks MTPC, 3/5 weeks MCS
11	Y	Y	6/7 weeks LDM, 8/8 weeks MTPC, 3/9 weeks MCS
14		Y	9/11 weeks LDM, 8/8 weeks MTPC, 3/5 weeks MCS
17			
19			
20			
21		Y	7/8 weeks LDM, 11/16 weeks MTPC
23			
24			
25		Y*	

* *Denote interviews that did not include text segments relevant to study aims and thus were not counted*.

### Participant Demographic Data

Most participants were non-Hispanic (*n* = 17; 94%), white (*n* = 17; 94%) males (*n* = 13; 72%) with an average age of about 47 years. Average lifetime opioid use was close to 10 years (range: 1–16). Most participants had more than one DSM-V diagnosis (89%), with two-thirds having an anxiety disorder (61%). Refer to Table 2 for a summary of baseline and demographic variables. The pilot subsample was reflective of the full feasibility/acceptability study sample for all categorical variables (e.g., gender and race); however, due to the small numbers, Fisher’s exact tests were not conducted.

**Table 2:** Demographic/baseline data.

Variable	Full Sample		Pilot Sample	
	(*n* = 18)		(*n* = 11)	
Male *N* (%)	13	(72.2)	9	(81.8)
Age (years), mean (SD)	47.4	(12.8)	48.3	(15.4)
Race, *N* (%)				
White	17	(94.4)	11	(100.0)
Other	1	(5.6)	0	(0.0)
Ethnicity Hispanic, *N* (%)	1	(5.6)	0	(0.0)
Lifetime Substance Use in Years, mean (SD)				
Opioids	6.8	(7.4)^1^	9.1	(8.0)
Cannabinoids	16.9	(14.9)^1^	18.7	(13.9)
Cocaine	9.5	(12.5)^1^	11.1	(13.9)
Alcohol	8.7	(11.1)^1^	10.7	(11.7)
ACE score 4+, *N* (%)	10	(55.6)^2^	5	(45.5)
Non-substance use DSM-V diagnoses, *N* (%)				
Single DSM-V diagnoses	2	(11.1)	1	(9.0)
2+ DSM-V diagnoses	16	(88.9)	10	(91.0)
Comorbid DSM-V diagnosis, *N* (%)				
Major depressive disorder	8	(44.4)	6	(54.5)
Anxiety disorder	11	(61.1)	6	(54.5)
PTSD	4	(22.2)	2	(18.2)
Have practice meditation before, *N* (%)	8	(44.4)	4	(36.4)

1 *Missing (*N* = 2)*.

2 *Missing (*N* = 1)*.

## Results

### Quantitative Findings: SCS-SF Scores

**Figure 3:**
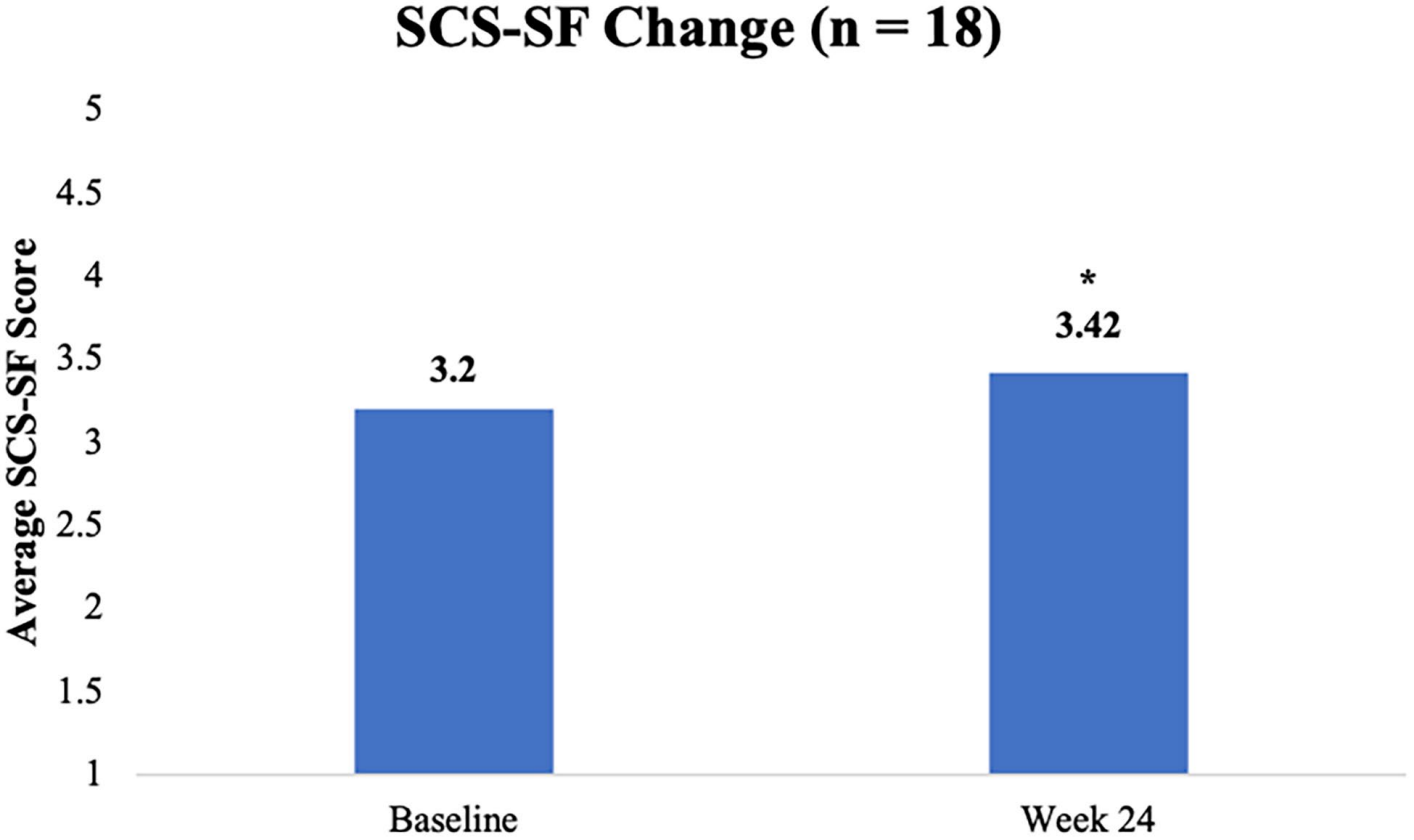
Change in SCS-SF scores from baseline to week 24. ^*^p < 0.05

**Figure 4:**
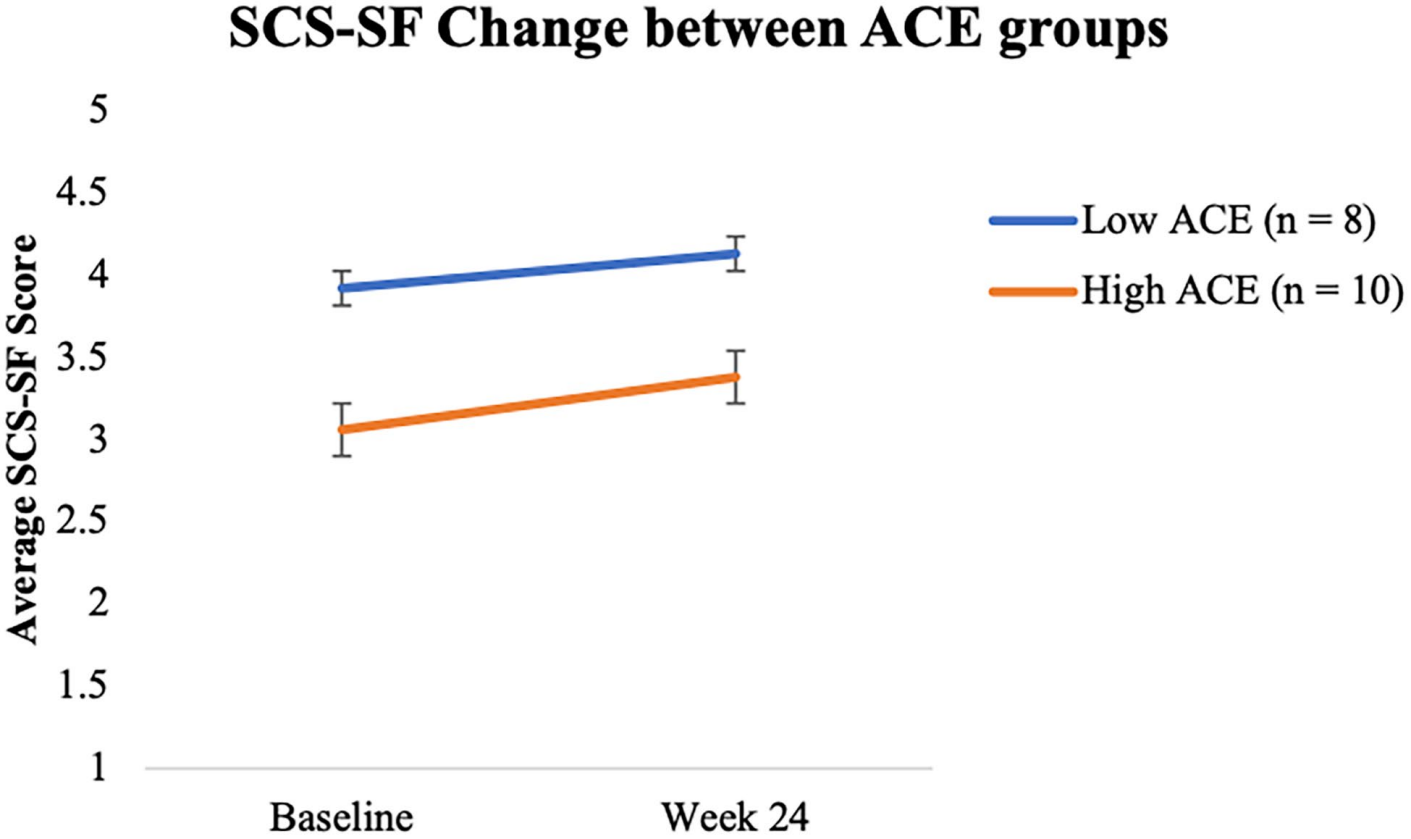
Change in SCS-SF over time between ACEs groups.

Mixed model analyses showed that, regardless of ACE score, all M-ROCC participants’ total average SCS-SF scores increased significantly from baseline to week 24 (*β* = 0.22, *p* = 0.028, *d* = 0.30; Refer to Figure 3). At baseline, participants with high ACE scores as compared to participants with low ACE scores tended to have lower levels of self-compassion with a large effect size difference, though it did not reach statistical significance (*β* = −0.82, *p* = 0.055, *d* = 1.09). From baseline to week 24, the self-compassion scores for the lower ACE group did not significantly increase (*β* = 0.19, *p* = 0.39, *d* = 0.25). However, self-compassion scores increased for the higher ACE group by week 24 (*β* = 0.25, *p* = 0.096, *d* = 0.34), but this also was not statistically significant (Refer to Figure 4). Despite the gains for the higher ACE group, the group differences did not significantly shrink between the ACE groups by week 24 (*β* = −0.75, *p* = 0.096, *d* = 1.00).

### Qualitative Findings: Interview data

Due to the analysis of a subset of the qualitative interview data, the following findings represent the voices of 11 participants whose interview transcripts included text coded relevant to study aims (Refer to Methods, Figure 5 for Participant Exposure to M-ROCC). Relevant text supporting the multidimensionality of self-compassion, operationalized by six SCS-SF subscales/factors, surfaced unevenly in the data. Participants attested to compassionate self-responding (i.e., mindfulness (19 instances), self-kindness (17 instances), and common humanity (4 instances) more often than they attested to uncompassionate self-responding [i.e., overidentification (8 instances), self-judgment (15 instances), and isolation (1 instance); Refer to Table 3 for exemplar quotes that provide evidence supporting subscale/factor representation in the data]. For those participants with data across two timepoints (*n* = 3; week 4—week 24), there was a shift in all three cases toward greater compassionate self-responding.

**Table 3:** Exemplar quotes: evidence supporting subscale representation in the dataset.

SCS-SF SUBSCALES	EXEMPLAR QUOTES
CSR	Mindfulness	*I’m working on getting my dentures. For some reason, I wasn’t mindful of not having them but now I’m mindful all the time of not having my teeth*. They got lost when I moved apartments somehow. I’ve been like six months without them and I’m very mindful of it. But before the group I wasn’t mindful. I did not care. So now I’m not [sic: read ‘I am’] mindful every day. And that’s helped me with that, and that’s part of my self-esteem also. [05: wk4]		What is it that I bring, the intelligence that I bring that exacerbates that self, that make it so, that elongates it, makes it worse, and *these moments of awareness is becoming definitely more common than they were* before. [01: wk24]
	Self-Kindness	[Can you tell me a bit about kindness toward yourself?] …I was using, and it was cocaine - Very afraid of my heart beating out of my chest and just a lot of fear, I was just, a lot of fear. And I shared with [staff] in the group that I used to simply place my hands on my chest like this when I was laying down with my eyes closed. I’d say with very simple intent, I’d say to myself “may healing relax and reiki energy flow through my hands until it’s needed in my body.” And I did this, I want to say hundreds of times. A lot. Dozens of times. And it’s, just talking about it right now it’s kind of funny how I would say it and I would say 9 out of 10 times if I was in that situation and it was usually when I felt the need to do so, [crosstalk 00:09:31] I probably would have gone and done it. And so, it was kind of like that God has strange ways of bringing you around to, *in that sense it was giving myself that kind of care and self-compassion. Healing intent maybe*. [01: wk4]
		I think with the meditation and allowing my whole self to calm down has allowed me to be like, “Girl, we are at three milligrams” [buprenorphine dose]. *I thought I would have been off by now but we are not. This is where we are and that’s okay too*. I keep that attitude instead of, “why cannot we just hurry it up.” I think the meditation has helped just to keep me calmer, which I realized that’s it’s okay, some things just take longer and that’s okay. [11: wk4]
	Common Humanity	But it amazes me, as long as we have been around, as long as I’ve been around, that somebody will say something or do something, and it’ll bother me. I do not say anything it’s just inside, “That really bothered me.” And then I think about it. “Oh yeah, of course it bothers. That’s your exclusion…”*Each time somebody tries to exclude you, you get really pissed. Who does not?* And he gets more angry when somebody’s trying to humiliate him. And so, it’s different for different people but I realize when that’s happening, I’m almost reliving that experience, emotionally. [14: wk24]
		And what’s best for me to do is keep my mouth shut first of all and then tell somebody about it. Because that helps me normalize myself and see it in perspective because when I go to these meetings, these little meetings and hear people talk about what’s going on in their day I say, “Wow, I feel much better because I do not feel so crazy anymore.” Because these are the things we do not talk about but when we talk about it, it really helped us. It helps us have empathy and compassion for each other and all of a sudden you realize, “*Yeah, everybody goes through that. It’s no big deal*. [14: wk24]
USR	Self-Judgment	I do feel guilty if I do not do it [meditate] when I say, I’m going to do it at the beginning of the day. [02: wk4]
		Well, when I was on all these drugs and doing all the crazy stuff, *I did not really care for myself. I just did not think I was going to get better*. [21: wk24]
	Overidentification	*With relapse, it’s helped me in the terms of not being so hard on myself and taking it slower*. Because when that did happen it would … I’d be like in a very anxious day. I’d be like, “Oh my gosh, what did I do?” It put me in a state of worrying, doubt and confusion all these feelings come up. And then I’d come in with [inaudible 00:40:28] we’d just practice the mindfulness. So that really helps. [02: wk4]
	Isolation	*When I’m alone, it’s very hard to be kind to myself* in general. [08: wk4]

*CSR = compassionate self-responding/USR = uncompassionate self-responding. The reader will find that some text segments could be coded in multiple ways given the multidimensionality of self-compassion and the interrelated nature of the constructs*.

#### Process: Disengagement in Uncompassionate Responding

Neff’s description of how one becomes more self-compassionate—through simultaneous engagement in self-compassionate responding and disengagement in uncompassionate responding (2020)—was substantiated in the data and expanded to include subthemes qualifying the process as a job to be done (e.g., “*task*”) [01/M: wk24], and the non-linearity, or “*back and forth*,” [04/F: wk4] of a behavior change process. Most participants described the process of disengagement from uncompassionate responding as challenging. In the following references to the challenges, study participants also offer several oft-repeated reasons for why becoming self-compassionate was so challenging. [Text is bolded in participant quotes below to draw the reader’s attention to language around a theme or to underscore the intersection of themes within quotes provided to substantiate a particular theme].

### Awareness That Self-Compassion Can Be Challenging, and Is Not Our Usual Way of Responding to Ourselves, Especially for Childhood Trauma Survivors

Though some participants did not express their challenges with the process, the following quotes highlight how most participants found it comparatively easier to criticize and judge themselves and engage in negative self-talk than it was to demonstrate self-compassion. It is noteworthy that 5/7 (71%) people who expressed challenges with self-compassion endorsed higher ACEs at baseline. For some, the challenge to becoming self-compassionate went so far as to seem impossible.

Can I accept myself as I am, just as I am? Sometimes it just **seems like such a heroic or insurmountable task**—like trying to jump over your own knees. [01/M: wk24]

Participants qualified the challenges they were experiencing in becoming more self-compassionate in terms of deep-seated, negative patterns of relating to themselves through allusions to difficult histories, and in some cases, specific references to trauma, substance use, and/or related shame. For example:

I think I just have a **big block** that I have to work with because every time I do it [practice self-kindness] I, A, recognize its importance and B, feel drawn to it. So, I think what’s behind there is some kind of **internalized negativity** toward myself that I need to disperse. I think it’s **been there for a while** otherwise it would not have so much power, and also, it’s still unknown to me. Only when we are talking about it do I start to realize that there’s some kind of **resistance** there. [14/M: wk24]

The struggles that many participants expressed regarding becoming self-compassionate suggest a starting point for future interventions in terms of heightening awareness around the expected nature of these challenges.

#### Process: Engagement in Compassionate Responding

Descriptions of compassionate responding coalesced around themes highlighting: (1) mindfulness as the foundational mechanism—or necessary skill—that potentiates directing compassion toward ourselves; (2) observations about kindness toward others as context for kindness toward ourselves; and 3) observations about common humanity emerge in the context of references to isolation and perceived exclusion.

### Mindfulness as Key Mechanism

References to the present moment and use of words like “*remind*” [11/F: wk24] (suggesting a return to focused attention/mindful awareness) surfaced throughout the interview data, underscoring mindfulness as the foundational mechanism that increases one’s ability to experience self-compassion. Despite challenges, mindfulness emerged repeatedly as the catalyst for change. For example:

…There's probably not a day that goes by where I don't think just a little negatively. Something will come up during the day, you know, I'll be in a situation. I can't remember everything, but I'll say, jeez, I wish I had been there for that or whatever. And that's when I'll go home and do that tape [mindfulness practice recording] and come back to where I should be, in the present**. There's no better place to be than the present.** [04/F: wk4]

[Interviewer: Any change in your kindness toward yourself in the period since we last talked?] …**I always try to remind** myself like, no matter what's going on, I'm doing better than I was yesterday…There's something I read one day that said, “tell yourself something positive, like, try not to say anything bad about yourself for like 10 days at all, like no matter what.” And so, I tried that, and I just kept **reminding myself** like for like 10 days, no matter what it was, just be kind to yourself and give yourself like a compliment almost…there were some days where I was like, I'm just not even going to say anything. I'm just going to **let self be**. [11/F, wk24]

Thus, the practical implications for intervention seem to suggest the primacy of teaching mindfulness prior to other self-compassionate skill sets.

### Outer kindness Leads to Observations About Inner or Self-Kindness

Participants Make observations about self-kindness in the context of references to kindness toward family members and others. Embedded in references to family members/others, and extending kindness, acceptance, and/or compassion to these people, lie references to mindfulness at work (“*I noticed*…,” [04/F: wk4]), as well as continued challenges, (“*kindness to myself is still lacking…that’s the hardest part*,” [05/M: wk4]) and insights into self-kindness (“*I’m just going to let self be”* [11/F: wk24]). Another participant noticed that he is better at not putting himself down (i.e., is kinder to himself), as a result of improved mood regulation (i.e., does not jump to conclusions or overreact) when faced with an upsetting situation with children and different obstacles of life.

**I noticed** now I don’t jump to conclusions, which is big for me. Especially regarding my daughters and different things in your life, where you’re confronted with something that… makes you kind of upset or mad or question yourself or the other person. My ability to stop, okay, don’t jump to… let it go for now, go back to it. Don’t rush into judgment of other people, either, or me…It’s a big thing for me… **for years, I did what I did, and I thought how I thought. I just think I’m a lot better at not putting myself down so much.** So quick to be, oh yeah, that was mean. I can think about it now. I can be more logical with it instead of acting on my emotions so much. [04/F: wk4]

I can get the kindness part toward other people and whatnot because I had that instilled in me from my upbringing. But the **kindness to myself is still lacking**. **That’s the hardest part.** [05/M: wk4]

Again, participants highlight potential guidance or practical implications for future interventions. Cultivation of kindness or a softness for others may be necessary scaffolding to support incremental steps toward self-kindness and maintaining ongoing commitment to regular mindfulness practice.

### Isolation or Perceived Exclusion Leads to Observations About Common Humanity

Common humanity is observed in the context of expressions of isolation and perceived exclusion. For the two participants who referenced common humanity, the context is revealing. Participant 10 speaks of “*stepping out of my little snow globe*,” [10/M: wk24] or less euphemistically, moving from being isolated to rejoining society through recovery. In that context, he notes his epiphany that all people have the right to live freely without judgment by others. Similarly, another participant speaks of feeling less “*crazy*,” [14/M: wk24] implying that he feels less alone in his feelings. This newfound self-compassion results from listening to other group members talk about “*things we do not talk about*,*”* [14/M: wk24] and coming to the realization that he is not so different or “other” (i.e., there is a common humanity).

I’m just trying to be more open and accepting and aware of everything that’s going on around me. Not just really in my own life, but the person next to me, all the people around me. Because **we all have to live in this world.** I guess I’m just kind of stepping out of my little snow globe that I’ve lived in for most of my life. [10/M: wk24]

…When I go to these meetings and hear people talk about what’s going on in their day I say, “Wow, I feel much better because I don’t feel so crazy anymore.” Because these are the things we don’t talk about but when we talk about it, it really helped us. **It helps us have empathy and compassion for each other and all of a sudden you realize, “Yeah, everybody goes through that. It’s no big deal.”** [14/M: wk24]

Participant 14 references a pattern of feeling excluded/rejected due to something “*somebody will say or do*,” but in recognizing (or being mindful) of this behavior pattern, he seems to express self-compassion in the casual phrase, “*who does not*?” [14/M: wk24]—a clear attestation to common humanity.

But it amazes me… somebody will say or do something, and it'll bother me. I don't say anything it's just inside, "that really bothered me." And then I think about it, "Oh yeah, of course it bothers. That's your exclusion." Each time somebody tries to exclude you, you get really pissed. **Who doesn't?** And he gets more angry when somebody's trying to humiliate him. [14/M: wk24]

Finally, participant 10—the participant who qualitatively seemed to reap the greatest benefit from the M-ROCC intervention based on interview data—shares another realization of common humanity while reflecting on his upbringing by “racist” parents. “*Everyone is everyone*,” he says. This realization, though “*a long time coming*,*”* seemed to empower him to look at himself in a new way and to say, “*that’s a genuinely good human being…that’s the person I’m turning into*.*”* [10/M: wk24]

All of those things I was a year ago [and] for all of my life, and now **I feel like I'm changing** … I guess if I saw myself from across the street, I'd like to say, that's a guy, that's a human being that's all right. That's a genuinely good human being. And I feel like that's the person that I'm turning into.

[So, you feel like a different person, and you don't think like you used to…what else do you mean by that?]

I can give you a perfect example…where I grew up, it was like 98% Caucasian, Roman-Catholic, so I was definitely raised as what I would now call racist. You just hear stories and stuff growing up, it was just normal talk…And I find myself now… just kind of shaking my head at myself, but knowing where that came from and just, without being mean to myself for thinking that way but I don't think that way at all anymore…

[And how does that feel to you now, this shift?]

I'm honestly still dealing with it because those words still come to my head … but **I'm far more mindful that I do it now**, which helps a lot. And **every time it makes me realize** I should just be nicer… **everyone is everyone**. You don't need to go there. And I think just that one example of the insight has a ripple effect to other behaviors and the way I had those, too. It's just more positive thinking and not negative thinking. And it's becoming automatic now. And the only really big change that has happened in my life has been the practice. [10/M: wk24]

This participant reflection is exemplary in that it illuminates the process of becoming more self-compassionate—simultaneous engagement in self-compassionate responding and disengagement in uncompassionate responding—taking the reader inside the black box of his exposure to M-ROCC and mindfulness specifically. [Notably, these data are from a week 24 interview and thus benefit from the participant perspective on the impact of the development of self-compassion over the time of the training]. He underscores mindfulness as a mechanism for his radical change (“those words still come to my head… but I’m far more mindful that I do it now”) but also highlights self-kindness (“without being mean to myself for thinking that way”). These revelations lead him to signal our common humanity (“everyone is everyone”), as well as the automaticity of more positive instead of negative thinking. Finally, the participant notes that this change in himself has had a “ripple effect” to other behaviors…evidence in support of a textbook description of how cognitive restructuring has the potential to generalize.

### M-ROCC Facilitated Changes in Self-Compassion “Tools in the Toolbox”

Almost half of the participants explicitly attributed their increased self-compassion (e.g., liking themselves) with their participation in the M-ROCC groups.

A lot of us addicts have a hard time forgiving ourselves or the self-love part of it. Today I can look in the mirror and like myself, but not love myself fully for my past deeds and whatnot…But that's gotten much better now. I go to all the parties. I talk to [family members] all the time…**that's all been since I started this group**. [05: wk4]

Other participants expressed that M-ROCC had helped with emotion regulation. For example, during the early years with multiple children under the age of five when patience is low and one can get “frustrated quickly” [10/M:wk4]. Another participant referenced that due to M-ROCC’s intensive MTPC-OUD group sessions, she had implemented a new morning routine in her home, prioritizing being less punitive (e.g., hurrying everyone out of bed) and instead beginning the day in a more positive and “upbeat” manner [11/F:wk4]. Generally, participants reported feeling more present and positive in their daily lives as a result of exposure to the intervention.

## Discussion

n this qualitative substudy, we sought elaboration of the finding that participants in our feasibility and acceptability study (Fatkin et al., 2021) made significant improvements in self-compassion from baseline to follow-up at week 24. Our aim in this exploratory analysis was to gain insights into the postulated mechanisms of action for self-compassion (i.e., self-kindness, mindfulness, and common humanity; absence of self-judgment, isolation, and overidentification) or how self-compassion enhances wellbeing, due to Neff’s theorizing that self-compassion is a dynamic system in which the components are in a “*synergistic state of interaction*” (Neff, 2016). Additionally, we sought to explore changes in self-compassion during the M-ROCC intervention among participants with “higher” (>/= 4) and “lower” (<4) ACEs at baseline to assess the differential effectiveness of the intervention due to its “trauma-informed” nature. Several findings warrant attention.

### M-ROCC May Increase Self-Compassion Among Patients With OUD

While the sample was not sufficiently powered to detect an effect in the quantitative data, disproportionate participant endorsement of self-kindness, mindfulness, and common humanity, compared with their inverses, suggest that M-ROCC may increase self-compassion by increasing compassionate self-responding and decreasing uncompassionate self-responding among patients with OUD during office-based opioid treatment (OBOT). A recent meta-analysis of randomized controlled trials investigating self-compassion interventions specifically examined the influence of group and individual models of delivery on self-compassion outcomes and found a stronger effect for group-based delivery. The authors suggested that group delivery enables a lived experience of connection (compared with isolation) and that groups encourage sharing/discussion which may help to reinforce common humanity and acceptance of personal flaws (Ferrari et al., 2019). This finding is corroborated by evidence that mindfulness therapies can increase self-compassion (e.g., Birnie et al., 2010; Gard et al., 2012; Greenberg et al., 2018; Gawande et al., 2019). Notably, in the present study, many participants explicitly attributed their increased self-compassion (e.g., liking themselves and less impulsive violence) to their participation in the M-ROCC groups, identifying their mindfulness-focused group experiences as the catalyst for change.

### Compassionate Self-Responding Challenges: Differential Effectiveness for Those With Higher ACEs

Paralleling pervasive references to CSR, most found self-compassion challenging—especially those participants who endorsed higher ACEs. Noted such difficulties are replete in the literature. In a study among people from the general population designed to gain knowledge on the interrelationships between compassion for others and self-compassion, participants strongly agreed that they felt compassion for others (*M* = 5.62 on 7-point scale), yet self-compassion (*M* = 3.07 on 7-point scale) was anchored at “sometimes” (López et al., 2018). The authors surmised that compassion toward others is consistent with the notion that it is evolutionarily determined (e.g., enhances welfare of vulnerable offspring, desirable emotion or attribute in mate selection processes, and because it enables cooperative relations with non-kin; Goetz et al., 2010) and thus is to be considered the norm rather than the exception. However, self-compassion is seemingly more elusive, like “*something that does not exist”* [01: wk. 4] (i.e., unless taught/learned behavior). In a study designed to explore the meaning and experiences of compassion and self-compassion for people with depression and anxiety (both conditions being pervasive among participants in our study), participants reflected that being self-compassionate would be difficult, either because the concept itself felt challenging to enact or their experience of psychological disorder had negatively impacted their ability to be self-compassionate (Pauley and McPherson, 2010).

Researchers have found that trauma (e.g., adverse childhood experiences) exposes people to unprecedented life events and that as a result they undergo processes of assimilation/consolidation and/or accommodation. During these processes, people may adopt extreme beliefs about the self (e.g., that they are undeserving of kindness; Gilbert et al., 2011). For example, childhood emotional maltreatment has been shown to promote the tendency for internalizing critical thinking toward the self, which becomes consolidated as part of the personality over time, and leads to the derailment of relationships in general, and romantic relationships in particular (Lassri and Shahar, 2012). Also, higher levels of childhood maltreatment have been demonstrated to lead mothers to hold more self-critical judgments in adulthood and to feel less efficacious in their role as mother (Michl et al., 2015). Thus, people exposed to trauma, and especially childhood maltreatment (Glassman et al., 2007; Joss et al., 2019), may perceive aspects of self-compassion to be incompatible with their new (“protective”) belief system and consequently may *actively resist* self-compassion because behaving compassionately would be in direct conflict with their schematic sense of self (Forkus et al., 2019). To underscore how challenging it may be for some to be self-compassionate, emerging evidence suggests that maltreatment alters trajectories of brain development representing actual differences in neural circuitry to affect sensory systems, threat detection, emotion regulation, and reward anticipation (Teicher et al., 2016).

Germer and colleagues (2009) noted that learning to respond to failure and feelings of inadequacy with kindness toward self instead of judgment can lead to a phenomenon called “*backdraft*.” Backdraft, or the confrontation with trauma histories and associated bottled-up emotions like anger or grief (Germer and Neff, 2019), has been observed among people in states of deprivation from love or compassion when they finally do open their hearts. This phenomenon was observed in our data. Most patients who expressed challenges with self-compassion endorsed higher ACEs at baseline.

Though exploratory, the data suggest differential potential importance of the “trauma-informed” intervention among those with childhood trauma, (Gawande et al., 2019). In other words, the tailoring of the intervention to those with trauma histories may have made it more salient, and with the potential to be more effective, for those who reported higher levels of adverse childhood experiences. This theory remains to be tested though and it is possible that a more focused intervention may accelerate and/or increase these effects. In support of tailoring to this population, a recent study (Phillips, 2019) enlisted latent profile analysis to examine how the different components of self-compassion interact *within* individuals to form self-compassion mindsets. Of the three mindsets identified (uncompassionate self-responding, moderately compassionate, and highly compassionate), the associations observed for those with an uncompassionate self-responding mindset (i.e., low levels of self-kindness represented the greatest predictor of psychopathological outcomes) are particularly relevant for our work. [For all three mindsets, although the self-compassion components appear to operate as a system, the components have different roles within each.] This observation supports tailoring to self-compassion driven interventions for demographic groups most likely to possess uncompassionate self-responding mindsets.

### Self-Compassion Mechanisms of Action: Mindfulness, Self-Kindness, and Common Humanity

Regarding self-compassion’s theorized mechanisms of action, observations regarding compassionate responding (i.e., mindfulness, self-kindness, and common humanity) emerged in our qualitative dataset. Despite challenges, mindfulness (*via* the M-ROCC intervention) emerged repeatedly and pervasively as the catalyst for behavior change with respect to self-compassion suggesting that it may be *the key mechanism* of action underlying the self-regulation outcome. Some level of mindfulness appears to be a necessary precondition to develop feelings of self-kindness and common humanity—as is evidenced in our data and interpreted further below. Neff suggested that mindfulness may lead to more common humanity because its de-identified nature takes attention away from the self (Neff, 2003a). More broadly, to understand relationships among the self-compassion subscales/factors, Dreisoerner et al. (2020) hypothesized that training mindfulness simultaneously improves the other components of self-compassion: “to learn effective strategies of dealing with emotional distress, unpleasant emotions first need to be noticed and acknowledged. Only then can they be addressed with kind behavior or be regarded as something all humans go through at times.” Our study findings supported this hypothesis.

The data related to self-kindness and common humanity provide support for the notion that some level of mindfulness appears to be a necessary precondition to develop these other mechanisms of self-compassion. Across references to emerging self-kindness and recognition of common humanity, language attesting to heightened awareness is found (i.e., “*moments of awareness*,*”* “*I noticed*,*” and “all of a sudden you realize”*). Beyond mindfulness as the necessary precondition, we found that attestations to self-kindness and other observations about self-kindness (e.g., its challenging nature) surfaced in the context of references to kindness toward family members and others. Research in social neuroscience provides increasing evidence that self and other are interconnected, both on a “conceptual and on an affective representational level,” (Trautwein et al., 2016) and that practices, such as loving kindness meditations (LKMs), may increase social connectedness (Hutcherson et al., 2008), empathy (Mascaro et al., 2013), and compassion (Klimecki et al., 2013, 2014), among other socially desirable feelings. In traditional LKMs (as well as the one used as part of M-ROCC), attention is directed toward *easier beings* at first (e.g., may you be happy and may you be healthy) with the expectation that one can learn to model/generalize kindness and compassion for ourselves and for more difficult relationships on the kindness and compassion we offer to loved ones/benefactors. Thus, expressions of/heightened awareness of kindness for others may precede expressions of/heightened awareness of self-kindness.

A limited number of studies have examined compassion for others together with self-compassion (e.g., Mongrain et al., 2011; Neff and Pommier, 2013). Notably, an fMRI study revealed that they involve similar brain regions (Longe et al., 2010) suggesting that people who are more compassionate toward others could certainly learn to be more compassionate or kind toward themselves. This may potentially be supported by a neuroimaging study that demonstrated reduced activation in the temporal parietal junction during experimental deep tissue pain, which is a brain region typically related to salience processing and the empathic witnessing of the experience of pain of others (Berry et al., 2020). Consistent with this finding, in a series of four experiments, Breines and Chen (2013) theorized that the experience of “giving support to another person may in turn increase the likelihood that people will take a supportive attitude toward themselves while support-giving schemas are activated” (p.59). They found that activating support-giving schemas increased self-compassion. Thus, practices that aim to cultivate self-kindness/direct loving kindness toward the self among self-critical populations (e.g., people in recovery) who are often deluged with internalized stigma and shame, may find it more efficient to encourage new practitioners to first focus attention on support for others with the aim to generalize to self (e.g., M-ROCC approach).

Finally, the two participants who referenced common humanity did so in the context of expressions of isolation and perceived exclusion or rejection, supporting Neff’s contention that the factors synergistically interact. In the context of an extended passage surfacing the essence of common humanity, one participant notes that increased mindfulness regarding his racist upbringing helped him to pay attention when racist thoughts occur and to think to himself, “*I should just be nicer…everyone is everyone…and the insight has a ripple effect to other behaviors”* (10: wk24). Although there is a clear theoretical link between mindfulness and moral reasoning or recognition of a common humanity (Rest, 1983), few studies have examined the effects of mindfulness practice on moral reasoning. Shapiro and colleagues examined the effect of mindfulness-based stress reduction (MBSR) on moral reasoning and decision-making. Two-month follow-up results showed that MBSR resulted in improvements in moral reasoning and ethical decision-making (Shapiro et al., 2012). Neuroscientific investigations have examined how mindfulness training or increased awareness of one’s thoughts following mindfulness training may lead to changes in how people process “*morally relevant information and potentially the promotion of moral action”* (Sevinc and Lazar, 2019). Though exploratory and an outlier in the dataset as one of the few 24-week interviews, it appears that participant #10’s experience with the impacts of increased mindfulness supports this effect, which may be something that emerges with ongoing practice.

### Practical Applications

In Crane et al.’s elegantly written editorial (2017) providing a framework to define the essential characteristics of the family of mindfulness-based programs (MBPs), they detail both the essential and variable elements of these programs. Given the high rates of self-criticism (Carlyle et al., 2019) and a proposed deficit of self-compassion in patients with OUD (Phelps et al., 2018), “warm” MBPs that integrate explicit threads of self-compassion throughout the mindfulness training (e.g., M-ROCC) may be particularly advantageous variable elements to complement buprenorphine treatment. This is especially true considering the lack of efficacy among alternative behavioral interventions (Carroll and Weiss, 2017). Practical applications of our work are derived from the results which suggest: (1) the need to heighten awareness that people with OUD experience challenges when cultivating self-compassion, (2) that mindfulness provides a key foundation for developing other self-compassionate skill sets, and (3) the cultivation of kindness or warmth toward others may serve as a first step or needed scaffolding to support incremental steps toward self-kindness. In terms of future work, elements of M-ROCC could be incorporated into other buprenorphine treatment programs, especially in primary care settings, to increase trauma-sensitivity and promote self-compassion. We plan to further examine the potentially mediating role of self-compassion in the effects of mindfulness on substance use, mental health outcomes, and levels of internalized stigma in a large well-powered randomized controlled trial.

### Limitations

This exploratory pilot may contribute to future studies with larger sample sizes and well-matched controls that have the statistical power to improve our understanding of how M-ROCC may increase compassionate self-responding, and decrease uncompassionate self-responding among patients with OUD with trauma histories. However, there were several notable limitations of the study. First, the small sample size precludes quantitative analyses with sufficient power to detect significant patterns or change. Second, the homogeneity of the sample is a limitation. Efforts to recruit more diverse samples in future studies designed to understand the effects of the program are warranted. Third, participants self-selected to participate in interviews which may have skewed the data more favorably toward the intervention (Teicher and Parigger, 2015). Fourth, while we did observe that there was a shift in all cases toward greater self-compassionate responding for those participants with data across two timepoints (*n* = 3; week 4-week 24), the smaller number of qualitative interviews at 24 weeks impeded a more complete understanding of changes in self-compassion over the extended data collection period for the full sample, and the ability to describe the developmental process of self-compassion over time during the intervention. That being said, pervasive references to CSR compared with USR after a mere 4 weeks post-exposure suggests M-ROCC’s enormous potential. Fifth, the use of the SCS-SF instead of the full self-compassion scale (SCS) limited operationalizing these outcomes using the SCS-SF subscales, as they are considered less reliable than the total score on the short form (Raes et al., 2011). While we favored the shorter form to reduce participant assessment burden, use of the longer form (SCS) in future studies will enable us to compare the subscale data to qualitative explorations, thereby increasing the potential for insights into mechanisms of action and relationships among self-compassion constructs. Sixth, the ACE questionnaire may not be the most specific measure to target the impact of childhood trauma or maltreatment. It is possible that examining the relationship between SCS and solely ACEs for abuse may produce a stronger signal and facilitate improved understanding of the underlying mechanisms at play. We plan to include additional scales in the future (Teicher and Parigger, 2015).

### Conclusion

M-ROCC may increase self-compassion among patients with OUD during OBOT by increasing compassionate self-responding and decreasing uncompassionate self-responding. Future studies should examine whether patients with OUD with childhood trauma (i.e., higher ACEs) may have lower levels of self-compassion, which could improve with M-ROCC. Future randomized controlled trials with larger, more diverse samples are needed to confirm these potential outcomes, mechanisms, and differential impacts between higher and lower ACEs subgroups.

## Data Availability Statement

The raw data supporting the conclusions of this article will be made available by the authors, without undue reservation.

## Ethics Statement

The studies involving human participants were reviewed and approved by Cambridge Health Alliance Institutional Review Board. The patients/participants provided their written informed consent to participate in this study.

## Author Contributions

ZS-O, RG, SM, and KO conceptualized the study. ZS-O, AF, and TF administered the study. SM conducted the qualitative interviews. SM and KO conducted the qualitative analyses and wrote the original draft. LS conducted the statistical analyses with TC’s supervision. ZS-O, AF, RG, TF, TC, and LS reviewed and edited the manuscript. All authors approved the final version of the manuscript for submission.

## Funding

This publication or project was made possible by Grant Number R21 AT010125 from the National Center for Complementary and Integrative Health (NCCIH) in collaboration with the National Institute on Drug Abuse (NIDA) through the HEAL initiative. Its contents are solely the responsibility of the authors and do not necessarily represent the official views of NCCIH, NIDA, or the National Institutes of Health.

## Conflict of Interest

The authors declare that the research was conducted in the absence of any commercial or financial relationships that could be construed as a potential conflict of interest.

## Publisher’s Note

All claims expressed in this article are solely those of the authors and do not necessarily represent those of their affiliated organizations, or those of the publisher, the editors and the reviewers. Any product that may be evaluated in this article, or claim that may be made by its manufacturer, is not guaranteed or endorsed by the publisher.
